# 2,3-Di­methyl­quinazolin-4(3*H*)-one

**DOI:** 10.1107/S1600536814013749

**Published:** 2014-06-18

**Authors:** Fozil E. Saitkulov, Azamat A. Tashniyazov, Azimjon A. Mamadrahimov, Kh. M. Shakhidoyatov

**Affiliations:** a Alisher Navoi Samarkand State University, Ministry of Higher and Secondary Special Education, University Avenue 15, Samarkand 703004, Uzbekistan; b Mirzo Ulugbek National University of Uzbekistan, Faculty of Chemistry, University St 6, Tashkent 100779, Uzbekistan; c Institute of Bioorganic Chemistry, Academy of Sciences of Uzbekistan, Mirzo Ulugbek St 83, Tashkent 100125, Uzbekistan; d S. Yunusov Institute of the Chemistry of Plant Substances, Academy of Sciences of Uzbekistan, Mirzo Ulugbek St, 77, Tashkent 100170, Uzbekistan

## Abstract

The non-H atoms of the title mol­ecule, C_10_H_10_N_2_O, are essentially coplanar, with a maximum deviation of 0.046 (4) Å for the O atom. In the crystal, mol­ecules are linked by weak C—H⋯O hydrogen bonds, forming chains along [010]. In addtion, weak C—H⋯π inter­actions and π–π stacking inter­actions between benzene and pyrimidine rings, with a centroid–centroid distance of 3.730 (3) Å, link the chains, forming a two-dimensional network parallel to (001).

## Related literature   

For the synthesis of related compounds, see: Takeuchi & Eguchi (1989 ▶). For the crystal structure of a related compound, see: Makhloufi *et al.* (2013 ▶). For standard bond lengths, see: Allen *et al.* (1987 ▶).
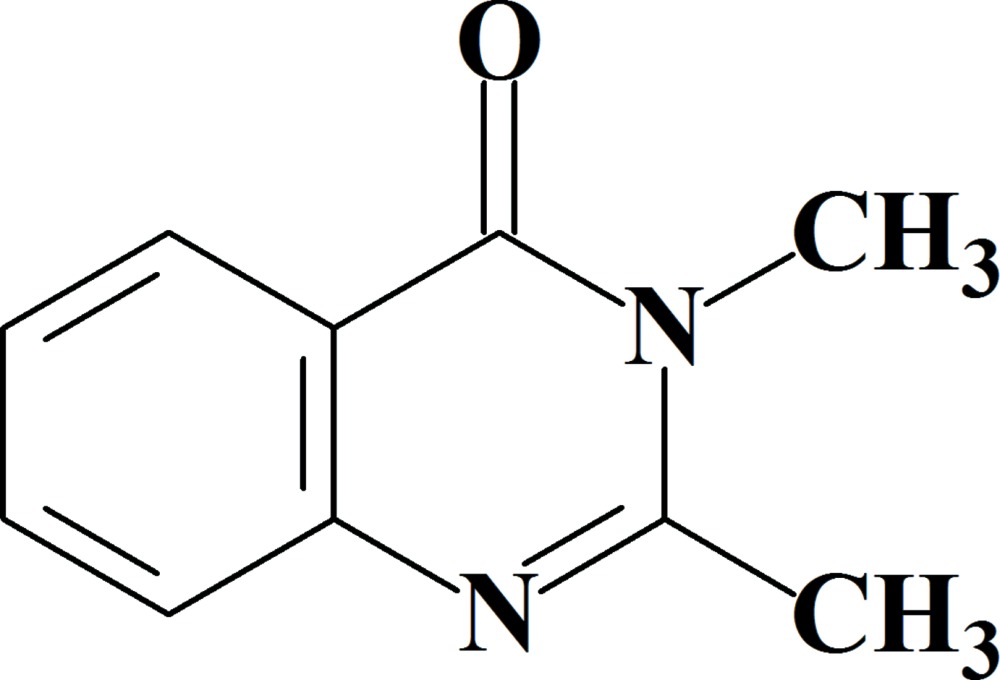


## Experimental   

### 

#### Crystal data  

C_10_H_10_N_2_O
*M* *_r_* = 174.20
Orthorhombic, 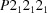

*a* = 4.826 (2) Å
*b* = 7.919 (3) Å
*c* = 23.060 (8) Å
*V* = 881.3 (11) Å^3^
*Z* = 4
Cu *K*α radiation
μ = 0.71 mm^−1^
*T* = 293 K
0.40 × 0.10 × 0.08 mm

#### Data collection  

Oxford Diffraction Xcalibur Ruby diffractometer
Absorption correction: multi-scan (*CrysAlis PRO*; Oxford Diffraction, 2009 ▶) *T*_min_ = 0.041, *T*_max_ = 1.000
2236 measured reflections
1585 independent reflections
821 reflections with *I* > 2σ(*I*)
*R*_int_ = 0.020

#### Refinement  

*R*[*F* ^2^ > 2σ(*F* ^2^)] = 0.071
*wR*(*F* ^2^) = 0.230
*S* = 0.97
1585 reflections
121 parameters
H-atom parameters constrained
Δρ_max_ = 0.20 e Å^−3^
Δρ_min_ = −0.19 e Å^−3^
Absolute structure: Flack (1983 ▶), 507 Friedel pairs
Absolute structure parameter: −0.3 (12)

### 

Data collection: *CrysAlis PRO* (Oxford Diffraction, 2009 ▶); cell refinement: *CrysAlis PRO*; data reduction: *CrysAlis PRO*; program(s) used to solve structure: *SHELXS97* (Sheldrick, 2008 ▶); program(s) used to refine structure: *SHELXL97* (Sheldrick, 2008 ▶); molecular graphics: *SHELXTL* (Sheldrick, 2008 ▶); software used to prepare material for publication: *publCIF* (Westrip, 2010 ▶).

## Supplementary Material

Crystal structure: contains datablock(s) I, GLOBAL. DOI: 10.1107/S1600536814013749/lh5714sup1.cif

Structure factors: contains datablock(s) I. DOI: 10.1107/S1600536814013749/lh5714Isup2.hkl

Supporting information file. DOI: 10.1107/S1600536814013749/lh5714Isup3.cml

CCDC reference: 1007927

Additional supporting information:  crystallographic information; 3D view; checkCIF report

## Figures and Tables

**Table 1:** Hydrogen-bond geometry (Å, °) *Cg* is the centroid of the N1/C2/N3/C4/C4*A*/C8*A* ring.

*D*—H⋯*A*	*D*—H	H⋯*A*	*D*⋯*A*	*D*—H⋯*A*
C10—H10*A*⋯O1^i^	0.96	2.47	3.345 (8)	151
C10—H10*B*⋯*Cg* ^ii^	0.96	2.80	3.608 (6)	142

Symmetry codes: (i) 
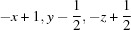
; (ii) 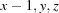.

**Fig. 1.**
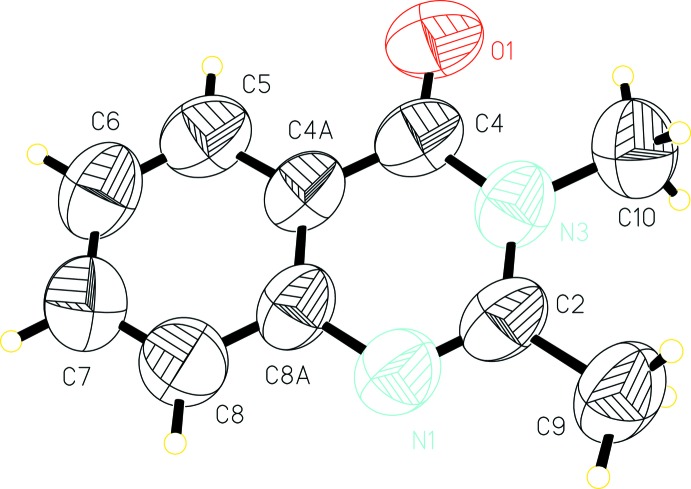
The molecular structure of the title compound with displacement ellipsoids are drawn at the 50% probability level.

**Fig. 2.**
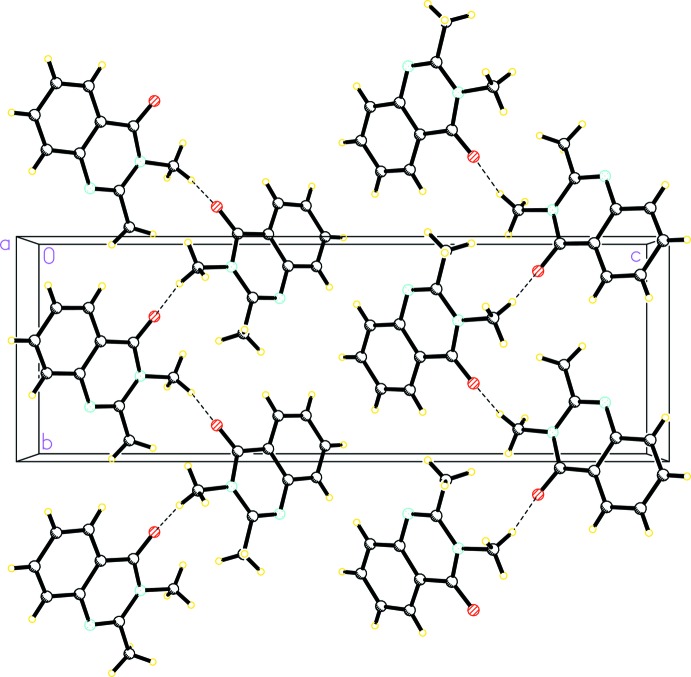
Crystal packing of the title compound showing a hydrogen bonds as dashed lines.

## supplementary crystallographic information

### S1. Comment

The molecular structure of the title compound is shown in Fig .1. The non-H atoms are essentially co-planar, with a maximum deviation of 0.046 (4) Å for atom O1. In the crystal, molecules are linked by weak C—H···O hydrogen bonds to form chains along [010] (Fig. 2). In addition, weak C—H···π interactions and π–π stacking interactions between benzene and pyrimidine rings with a centroid–centroid distance of 3.730 (3)Å, link chains forming a two-dimensional network parallel to (001). The bond distances (Allen *et al.*, 1987) and angles are in normal ranges. The crystal structure of a related cation is reported in the literature (Makhloufi *et al.*, 2013) and the synthesis of compounds related to the title compound is described by (Takeuchi & Eguchi, 1989).

### S2. Experimental

2-Methylquinazolin-4-one (0.01) mol was disolved in 45 ml absolute ethanol, then 2.5 mmol of NaH was added and then shaken for 30 min. To the reaction mixture was added solution of 0.01 mol methyliodide in 5 ml ethanol and the reaction mixture was refluxed for 4 h at 363 K. To this mixture was added 100 ml of cold water and then extracted with chloroform. The title compound was obtained in 69% yield with m.p. 491 K. Crystals suitable for X-ray analysis were obtained by slow evaporation of a solution of the title compound in ethanol.

### S3. Refinement

Carbon-bound H atoms were placed geometrically and treated as riding on their parent atoms, with C—H distances of 0.93 Å (aromatic) and 0.96 Å (methyl) and were refined with *U*_iso_(H)=1.2U_eq_(C) for aromatic and *U*_iso_(H)=1.5U_eq_(C) for methyl H atoms.

### Crystal data

**Table:**

C_10_H_10_N_2_O	*D*_x_ = 1.313 Mg m^−^^3^
*M**_r_* = 174.20	Melting point: 491(2) K
Orthorhombic, *P*2_1_2_1_2_1_	Cu *K*α radiation, λ = 1.54184 Å
Hall symbol: P 2ac 2ab	Cell parameters from 333 reflections
*a* = 4.826 (2) Å	θ = 3.8–64.0°
*b* = 7.919 (3) Å	µ = 0.71 mm^−^^1^
*c* = 23.060 (8) Å	*T* = 293 K
*V* = 881.3 (11) Å^3^	Needle, colourless
*Z* = 4	0.40 × 0.10 × 0.08 mm
*F*(000) = 368	

### Data collection

**Table:**

Oxford Diffraction Xcalibur Ruby diffractometer	1585 independent reflections
Radiation source: fine-focus sealed tube	821 reflections with *I* > 2σ(*I*)
Graphite monochromator	*R*_int_ = 0.020
Detector resolution: 10.2576 pixels mm^-1^	θ_max_ = 75.9°, θ_min_ = 3.8°
ω scans	*h* = −5→5
Absorption correction: multi-scan (*CrysAlis PRO*; Oxford Diffraction, 2009)	*k* = −5→9
*T*_min_ = 0.041, *T*_max_ = 1.000	*l* = −28→28
2236 measured reflections	

### Refinement

**Table:**

Refinement on *F*^2^	Secondary atom site location: difference Fourier map
Least-squares matrix: full	Hydrogen site location: inferred from neighbouring sites
*R*[*F*^2^ > 2σ(*F*^2^)] = 0.071	H-atom parameters constrained
*wR*(*F*^2^) = 0.230	*w* = 1/[σ^2^(*F*_o_^2^) + (0.1116*P*)^2^] where *P* = (*F*_o_^2^ + 2*F*_c_^2^)/3
*S* = 0.97	(Δ/σ)_max_ < 0.001
1585 reflections	Δρ_max_ = 0.20 e Å^−^^3^
121 parameters	Δρ_min_ = −0.19 e Å^−^^3^
0 restraints	Absolute structure: Flack (1983), 507 Friedel pairs
Primary atom site location: structure-invariant direct methods	Absolute structure parameter: −0.3 (12)

### Special details

**Table:**

Geometry. All e.s.d.'s (except the e.s.d. in the dihedral angle between two l.s. planes) are estimated using the full covariance matrix. The cell e.s.d.'s are taken into account individually in the estimation of e.s.d.'s in distances, angles and torsion angles; correlations between e.s.d.'s in cell parameters are only used when they are defined by crystal symmetry. An approximate (isotropic) treatment of cell e.s.d.'s is used for estimating e.s.d.'s involving l.s. planes.
Refinement. Refinement of *F*^2^ against ALL reflections. The weighted *R*-factor *wR* and goodness of fit *S* are based on *F*^2^, conventional *R*-factors *R* are based on *F*, with *F* set to zero for negative *F*^2^. The threshold expression of *F*^2^ > σ(*F*^2^) is used only for calculating *R*-factors(gt) *etc*. and is not relevant to the choice of reflections for refinement. *R*-factors based on *F*^2^ are statistically about twice as large as those based on *F*, and *R*- factors based on ALL data will be even larger.

### Fractional atomic coordinates and isotropic or equivalent isotropic displacement parameters (Å^2^)

**Table:**

	*x*	*y*	*z*	*U*_iso_*/*U*_eq_	
N3	0.3333 (9)	−0.1245 (5)	0.17512 (17)	0.0819 (12)	
O1	0.4020 (9)	0.1511 (4)	0.19869 (17)	0.1055 (13)	
N1	0.5799 (9)	−0.2643 (5)	0.10074 (17)	0.0846 (11)	
C4A	0.6580 (10)	0.0357 (6)	0.1199 (2)	0.0773 (13)	
C4	0.4620 (11)	0.0295 (7)	0.1667 (2)	0.0807 (13)	
C10	0.1309 (12)	−0.1350 (7)	0.2233 (2)	0.1009 (17)	
H10A	0.2040	−0.2059	0.2534	0.151*	
H10B	−0.0397	−0.1819	0.2093	0.151*	
H10C	0.0975	−0.0240	0.2386	0.151*	
C8	0.8983 (11)	−0.1088 (7)	0.0431 (2)	0.0912 (15)	
H8	0.9313	−0.2067	0.0219	0.109*	
C2	0.4000 (11)	−0.2650 (6)	0.1422 (2)	0.0816 (13)	
C8A	0.7099 (9)	−0.1120 (6)	0.0883 (2)	0.0769 (12)	
C7	1.0368 (13)	0.0367 (8)	0.0292 (2)	0.1023 (16)	
H7	1.1647	0.0374	−0.0010	0.123*	
C9	0.2541 (14)	−0.4274 (7)	0.1551 (3)	0.109 (2)	
H9A	0.2779	−0.4552	0.1954	0.164*	
H9B	0.3305	−0.5158	0.1316	0.164*	
H9C	0.0603	−0.4153	0.1468	0.164*	
C6	0.9842 (12)	0.1838 (7)	0.0607 (3)	0.1019 (18)	
H6	1.0776	0.2828	0.0514	0.122*	
C5	0.7965 (12)	0.1837 (6)	0.1051 (2)	0.0923 (16)	
H5	0.7615	0.2828	0.1255	0.111*	

### Atomic displacement parameters (Å^2^)

**Table:**

	*U*^11^	*U*^22^	*U*^33^	*U*^12^	*U*^13^	*U*^23^
N3	0.077 (2)	0.080 (3)	0.089 (3)	0.004 (2)	0.003 (2)	0.002 (2)
O1	0.118 (3)	0.083 (2)	0.115 (3)	0.008 (2)	−0.002 (3)	−0.024 (2)
N1	0.080 (2)	0.080 (2)	0.094 (3)	−0.004 (2)	0.002 (2)	−0.005 (2)
C4A	0.074 (3)	0.072 (3)	0.086 (3)	−0.001 (2)	−0.011 (3)	−0.001 (3)
C4	0.083 (3)	0.073 (3)	0.086 (3)	0.010 (3)	−0.011 (3)	−0.004 (3)
C10	0.100 (4)	0.104 (4)	0.098 (3)	0.020 (4)	0.012 (3)	0.008 (3)
C8	0.085 (3)	0.090 (3)	0.099 (3)	0.004 (3)	0.003 (3)	−0.012 (3)
C2	0.076 (3)	0.066 (3)	0.102 (3)	0.004 (3)	−0.006 (3)	−0.002 (3)
C8A	0.068 (3)	0.071 (3)	0.091 (3)	−0.001 (3)	−0.005 (3)	0.003 (3)
C7	0.092 (4)	0.117 (4)	0.098 (3)	−0.003 (4)	0.006 (3)	0.006 (4)
C9	0.103 (4)	0.077 (3)	0.147 (5)	−0.002 (3)	0.014 (4)	0.002 (4)
C6	0.096 (4)	0.092 (4)	0.118 (4)	−0.013 (3)	−0.008 (4)	0.022 (3)
C5	0.093 (4)	0.076 (3)	0.108 (4)	0.003 (3)	−0.009 (3)	−0.002 (3)

### Geometric parameters (Å, º)

**Table:**

N3—C4	1.382 (6)	C8—C7	1.370 (7)
N3—C2	1.385 (6)	C8—C8A	1.383 (7)
N3—C10	1.482 (6)	C8—H8	0.9300
O1—C4	1.248 (6)	C2—C9	1.496 (7)
N1—C2	1.292 (6)	C7—C6	1.397 (8)
N1—C8A	1.390 (6)	C7—H7	0.9300
C4A—C5	1.392 (7)	C9—H9A	0.9600
C4A—C8A	1.400 (6)	C9—H9B	0.9600
C4A—C4	1.436 (7)	C9—H9C	0.9600
C10—H10A	0.9600	C6—C5	1.368 (7)
C10—H10B	0.9600	C6—H6	0.9300
C10—H10C	0.9600	C5—H5	0.9300
			
C4—N3—C2	121.8 (4)	N1—C2—C9	117.9 (5)
C4—N3—C10	116.8 (4)	N3—C2—C9	118.2 (5)
C2—N3—C10	121.3 (5)	C8—C8A—N1	117.9 (5)
C2—N1—C8A	117.4 (4)	C8—C8A—C4A	119.6 (5)
C5—C4A—C8A	119.4 (5)	N1—C8A—C4A	122.4 (4)
C5—C4A—C4	121.9 (5)	C8—C7—C6	119.4 (5)
C8A—C4A—C4	118.7 (5)	C8—C7—H7	120.3
O1—C4—N3	119.5 (5)	C6—C7—H7	120.3
O1—C4—C4A	124.9 (5)	C2—C9—H9A	109.5
N3—C4—C4A	115.6 (4)	C2—C9—H9B	109.5
N3—C10—H10A	109.5	H9A—C9—H9B	109.5
N3—C10—H10B	109.5	C2—C9—H9C	109.5
H10A—C10—H10B	109.5	H9A—C9—H9C	109.5
N3—C10—H10C	109.5	H9B—C9—H9C	109.5
H10A—C10—H10C	109.5	C5—C6—C7	120.7 (5)
H10B—C10—H10C	109.5	C5—C6—H6	119.7
C7—C8—C8A	120.8 (5)	C7—C6—H6	119.7
C7—C8—H8	119.6	C6—C5—C4A	120.1 (5)
C8A—C8—H8	119.6	C6—C5—H5	120.0
N1—C2—N3	124.0 (5)	C4A—C5—H5	120.0

### Hydrogen-bond geometry (Å, º)

Cg is the centroid of the N1/C2/N3/C4/C4A/C8A ring.

**Table:**

*D*—H···*A*	*D*—H	H···*A*	*D*···*A*	*D*—H···*A*
C10—H10*A*···O1^i^	0.96	2.47	3.345 (8)	151
C10—H10*B*···*Cg*^ii^	0.96	2.80	3.608 (6)	142

Symmetry codes: (i) −*x*+1, *y*−1/2, −*z*+1/2; (ii) *x*−1, *y*, *z*.
